# Secondary Restless Legs Syndrome during psychopharmacological treatment: real-world evidence from a multinational pharmacovigilance program

**DOI:** 10.1093/ijnp/pyag029

**Published:** 2026-06-07

**Authors:** Gernot Fugger, Ulrich Rabl, Lucie Bartova, Valentin Popper, Victoria Watzal, Dominik Ivkic, Jakob Donath, Constance Busvine, Waldemar Greil, Sermin Toto, Renate Grohmann, Richard Frey, Anastasios Konstantinidis, Andreas Erfurth, Siegfried Kasper

**Affiliations:** Department of Psychiatry and Psychotherapeutic Medicine, University Hospital St. Pölten, Karl Landsteiner Private University of Health Sciences, Krems an der Donau, Austria; Department of Psychiatry and Psychotherapy, Medical University of Vienna, Vienna, Austria; Department of Psychiatry and Psychotherapeutic Medicine, University Hospital St. Pölten, Karl Landsteiner Private University of Health Sciences, Krems an der Donau, Austria; Department of Psychiatry and Psychotherapy, Medical University of Vienna, Vienna, Austria; Comprehensive Center for Clinical Neurosciences and Mental Health, Medical University of Vienna, Vienna, Austria; Department of Psychiatry and Psychotherapy, Medical University of Vienna, Vienna, Austria; Comprehensive Center for Clinical Neurosciences and Mental Health, Medical University of Vienna, Vienna, Austria; Department of Psychiatry and Psychotherapy, Medical University of Vienna, Vienna, Austria; Comprehensive Center for Clinical Neurosciences and Mental Health, Medical University of Vienna, Vienna, Austria; Department of Psychiatry and Psychotherapy, Medical University of Vienna, Vienna, Austria; Comprehensive Center for Clinical Neurosciences and Mental Health, Medical University of Vienna, Vienna, Austria; Department of Psychiatry and Psychotherapy, Medical University of Vienna, Vienna, Austria; Comprehensive Center for Clinical Neurosciences and Mental Health, Medical University of Vienna, Vienna, Austria; Department of Psychiatry and Psychotherapy, Medical University of Vienna, Vienna, Austria; Comprehensive Center for Clinical Neurosciences and Mental Health, Medical University of Vienna, Vienna, Austria; Department of Psychiatry and Psychotherapeutic Medicine, University Hospital St. Pölten, Karl Landsteiner Private University of Health Sciences, Krems an der Donau, Austria; Psychiatric Private Hospital, Sanatorium Kilchberg, Zurich, Switzerland; Department of Psychiatry and Psychotherapy, LMU University Hospital, Munich, Germany; Department of Psychiatry, Social Psychiatry and Psychotherapy, Hannover Medical School, Hannover, Germany; Department of Psychiatry and Psychotherapy, LMU University Hospital, Munich, Germany; Department of Psychiatry and Psychotherapy, Medical University of Vienna, Vienna, Austria; Comprehensive Center for Clinical Neurosciences and Mental Health, Medical University of Vienna, Vienna, Austria; Department of Psychiatry and Psychotherapy, Medical University of Vienna, Vienna, Austria; BBRZMed Simmering, Center for Mental Health, Vienna, Austria; 1st Department of Psychiatry and Psychotherapeutic Medicine, Hietzing Hospital, Vienna, Austria; Karl Landsteiner Institute for Clinical Risk Management, Karl Landsteiner Society, Vienna, Austria; Department of Psychiatry and Psychotherapy, Medical University of Vienna, Vienna, Austria; Comprehensive Center for Clinical Neurosciences and Mental Health, Medical University of Vienna, Vienna, Austria; Center for Brain Research, Medical University of Vienna, Vienna, Austria

**Keywords:** Drug-Related Side Effects and Adverse Reactions, Psychopharmacology, Antidepressive Agents, Antipsychotic Agents, Restless Legs Syndrome

## Abstract

**Objective:**

Secondary Restless Legs Syndrome (RLS) has repeatedly been associated with psychopharmacotherapy, especially antidepressants and antipsychotics. However, existing evidence is conflicting in terms of the magnitude of the association as well as the significance of substance classes or individual compounds. The objective of the present study was to evaluate secondary RLS linked to psychotropic medication in real-world, inpatient clinical routine treatment settings.

**Methods:**

A large dataset from a multinational pharmacovigilance program in German-speaking countries (*Arzneimittelsicherheit in der Psychiatrie*, “Drug safety in Psychiatry”) from January 2001 to December 2016 was retrospectively analyzed.

**Results:**

In a total of 340 099 monitored inpatients, 67 cases of newly diagnosed and severe RLS related to psychotropic drug treatment were recorded equivalent to a relative frequency of 0.02%. Over 80% of the cases were attributable to two compounds: the antidepressant mirtazapine and the antipsychotic quetiapine. Mirtazapine was found in 39 patients, while quetiapine was found in 16 patients with secondary RLS. For both substances, drug dosages were generally low at onset of secondary RLS. Most cases exhibited an onset within 1 or 2 days after dosage start or change.

**Conclusion:**

Histamine neurotransmission may represent a crucial common denominator in terms of secondary RLS in association with psychopharmacotherapeutics, as both substances exhibit antihistamingeric effects at lower dosages.

**Significance statement**

Restless Legs Syndrome (RLS) is a condition that causes a strong urge to move the legs, particularly at night. Medications used to treat depression and other mental health conditions have been linked to RLS, but it is unclear which medications are most likely involved. Our study used data from 340 099 psychiatric hospital inpatients. We found that severe secondary RLS caused by these medications was rare. Most cases happened in patients taking low doses of the antidepressant mirtazapine or the antipsychotic quetiapine. At low doses, both these medications block histamine receptors. This suggests that histamine neurotransmission may play an important role in the development of secondary RLS.

## Introduction

Restless Legs Syndrome (RLS) is a sensorimotor disorder that typically occurs or worsens during the evening or night while at rest. In summary, the diagnostic criteria include an urge to move the limbs during rest or inactivity accompanied or triggered by discomfort in the latter, whereby movement typically results in temporary symptom relief.^1^ Prevalence in population-based studies of the disorder tend to vary with rates between 5% and 13% in European and North American samples.^2^ The pathogenetic model of RLS is not fully understood, but iron deficiency in the brain with the induction of dopamine dysregulation and a hyperglutamatergic state may be related to sensorimotor and sleep symptoms.^3^ In addition, there is a classification of idiopathic RLS in contrast to symptomatic or secondary forms that are typically associated with pregnancy, iron deficiency, end stage renal disease requiring hemodialysis as well as other neurologic, cardiovascular or rheumatologic disorders, and different types of medication.^3^^,^^4^ There is compelling evidence about higher rates of RLS in psychiatric populations, especially in patients with mood disorders.^5^ Psychopharmacotherapeutics, especially antidepressants (ADs), were repeatedly found to be associated with the onset or the worsening of RLS in case reports, case series, observational, and controlled studies.^5^ Findings from systematic reviews and meta-analyses dedicated to this topic exhibited substantial heterogeneity and tended to be partially conflicting regarding effect sizes and compounds.^6^^,^^7^ To narrow this knowledge gap, we sought to evaluate secondary RLS associated with psychotropic medication in real-world clinical routine treatment settings of psychiatric inpatients by analyzing a large dataset from a multinational pharmacovigilance program.

## Methods

### Experimental procedures

The retrospective analysis of RLS symptoms during psychopharmacological treatment was based on data obtained from the AMSP (*Arzneimittelsicherheit in der Psychiatrie*, “Drug safety in Psychiatry”), which is a continuous observational and noninterventional post-marketing drug surveillance program in German-speaking countries (Germany, Austria, Switzerland). The objective of AMSP is to record severe or rare adverse drug reactions (ADRs) occurring during inpatient routine clinical care. Adverse events resulting from inefficiency or intentional intoxication are not included in the AMSP database.

Regarding methodological details of AMSP, we refer to previous work^8–10^ and provide the most important key points of the program hereinafter. Trained medical doctors act as drug monitors at their local psychiatric institutions. They discuss severe ADRs with treating physicians and ensure adequate documentation of cases by employing a standardized questionnaire, including a detailed description of the ADR and the drug treatment, as well as clinical and demographic data. Additional data documented in the AMSP questionnaire refer to potential risk factors, alternative explanations for the emergence of the ADR as well as considerations in regards to differential diagnosis and treatment of the ADR. Each documented case is first reviewed by local senior psychiatrists. Then, cases are discussed at regional and central case conferences, which take place twice a year, whereby officials from national authorities for regulating drugs and representatives from the pharmaceutical industry are also invited to participate.

Based on the study guidelines of the project,^9^ each ADR is subject to a probability rating. In detail, ADRs are graded 1 to 4 according to the information available, whereby an ADR is graded 1 or “*possible*” if the risk of an ADR is not known for the drug or the probability of another cause is considered to be >50%. A grade 2 rating is categorized as a “*probable*” ADR, which means the ADR is known for the drug, the time course and dosage are in accordance with previous experience, and alternative explanations are less likely. ADRs are graded 3 or “*definite*” when a re-exposure to a drug causes the same ADR in addition to the criteria for grade 2. An ADR is classified as grade 4 if it is considered “*questionable*” or “*not sufficiently documented*”—such as when an ADR is unusual and an alternative explanation is more likely, making the ADR improbable but not definitively excludable.

Furthermore, the AMSP data provide information on whether an ADR is attributable to a substance alone or in combination with other drugs. The assessment of relative frequencies is provided, hence, additional data on patients exposed to psychotropic drugs at each participating treatment center are collected twice each year on so-called reference days. The prescription pattern of all inpatients is recorded, including information on dosage together with basic information on sociodemographic variables and diagnoses. The number of inpatients and the mean treatment duration for all patients under surveillance per year is also provided by all participating centers.

The pharmacovigilance project AMSP received authorization by the leading boards of the participating institutions. Evaluations based on the AMSP database were approved by the Ethical Committees of the University of Munich and the Hannover Medical School (No. 8100_BO_S_2018) as well as the Medical University of Vienna (EK No. 1813/2020).

The current retrospective study analyzed reported cases of secondary RLS from 99 psychiatric hospitals located in Austria, Germany, and Switzerland. Data collection took place between January 2001 and December 2016. Based on the AMSP study guidelines,^9^ secondary RLS was diagnosed in accordance with the diagnostic criteria of the “International Restless Legs Syndrome Study Group.”^11^ Minimal requirement of ADR assessment was the prescription of at least one psychoactive drug with a causal relationship rated as “probable” or “definite.” Generally, severe ADRs in the study were defined as (potentially) life-threatening or seriously endangering the patient’s health, requiring the patient’s transfer to another department or ward providing more intensive care, or considerably impairing everyday functioning. Hence, in order to be considered a severe ADR in the present report, RLS had to exert a severe impairment on the patient’s well-being (eg, sleepless nights because of RLS). In addition to the documentation of the essential criteria for the diagnosis of RLS and the prior history of the disease before drug initiation, other secondary causes such as iron deficiency, renal failure, peripheral neuropathy, excessive alcohol, caffeine, or tobacco use were ruled out. In some of the cases, a polysomnogram was additionally administered to assess the presence of periodic leg movements during sleep.

Drugs with less than 5000 exposed patients within the total study sample were excluded. This applied to agomelatine, bupropion, chloral hydrate, clomethiazole, clomipramine, clonazepam, fluoxetine, flupentixol decanoate, fluphenazine, fluvoxamine, haloperidol decanoate, imipramine, maprotiline, mianserine, moclobemide, nortriptyline, oxcarbazepine, reboxetine, risperidone (long-acting injectable), ziprasidone, zotepine, zuclopenthixol acetate, and zuclopenthixol decanoate (alphabetical order). The threshold of 5000 was chosen as a conservative benchmark used in prior analyses of AMSP data to allow reliable detection of event rates up to 1 per 1000 exposures with an upper 95% confidence limit.^10^^,^^12^

### Statistics

The total population and subgroup frequencies were described using absolute and relative frequencies, with results expressed as percentages where appropriate. Between-group comparisons were conducted using chi-square tests (χ^2^) to assess whether differences in RLS incidence were statistically significant. Fisher’s exact test was applied in cases where any expected frequency in a contingency table fell below five. A significance threshold of α = .05 (two-tailed) was used to determine statistical significance. RLS incidences for drug groups or specific drugs are presented separately for cases where the drug is considered the sole cause (“attributed alone”) and for cases where it is part of a combination of drugs (“attributed alone and in combination,”) with the analysis restricted to cases rated “probable” or “definite.” Briefly, an ADR is classified as “attributed alone” for a given medication if that medication is rated as “probable” or “definite,” and no other medication in the same case is similarly implicated with a probability grade of “probable” or higher (accordingly, drugs co-attributed with the lowest rating, “possible,” are excluded). The “attributed alone and in combination” category includes all cases rated grade 2 (probable) or grade 3 (definite), even those where other drugs are equally likely. Therefore, individual cases may be counted for multiple drugs, and the total number of cases across all drugs may exceed the actual number of unique cases. All individual cases are presented in tabular form in the supplementary material.

**Figure 1:**
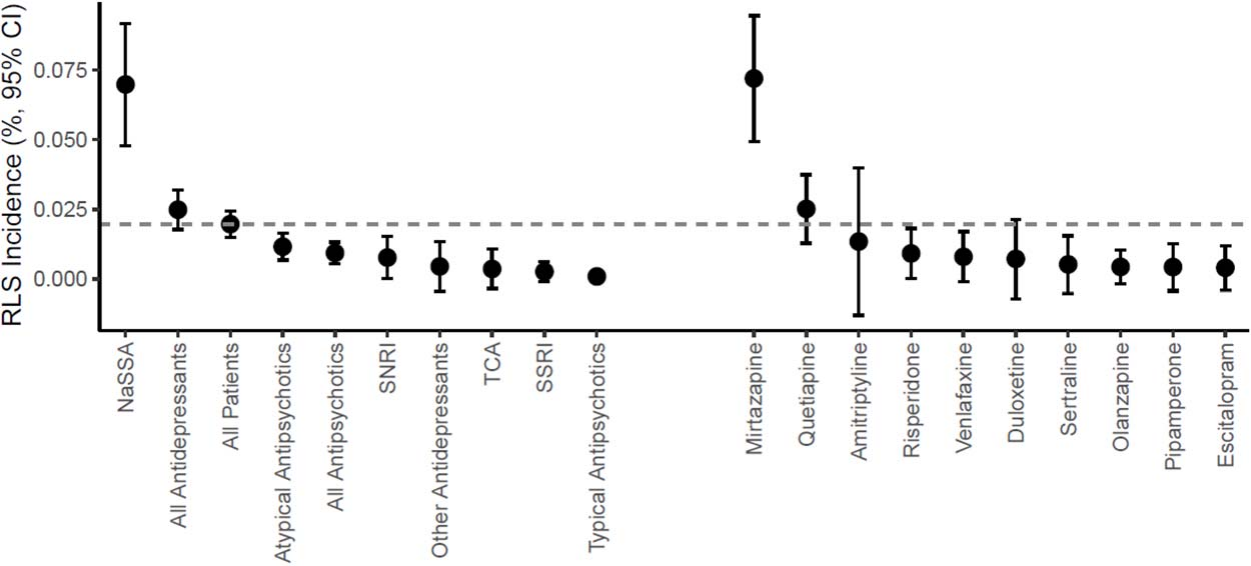
Incidence rates (±95% confidence intervals) of RLS (imputed alone) during treatment with psychotropic drugs. Dashed line indicates mean level of RLS for all patients.

For Figure 1, the percentages of cases imputed alone across different drug groups and specific drugs were displayed along with their 95% confidence intervals (95% CI). The standard error and confidence intervals were derived using the standard formula for proportions. A horizontal dashed line represents the mean percentage across all patients for reference. R version 4.4.1 (2024-06-14) was used for all analyses, employing the tidyverse, stats, kableExtra, and knitr packages for analyses, table, and figure preparation.

## Results

### Demographic factors

Among 340 099 patients treated during the observation period, 67 cases (0.02%) of secondary RLS were documented (Table 1). The incidence was slightly higher among patients under 65 years of age (61/266 473; 0.02%) compared to those aged 65 or older (6/73 626; 0.01%; χ^2^(1) = 5.639, *P* = .018). RLS occurrence did not differ significantly by sex (male: 25/151 066, 0.02%; female: 42/189 033, 0.02%; χ^2^(1) = 1.097, *P* = .295).

**Table 1:** Demographic and clinical characteristics of the sample.

Group	RLS cases/*n* exposed	Percentage (%)	Statistics
All patients	67/340 099	0.02	
Age, <65 years	61/266 473	0.02	χ^2^(1) = 5.639, *P* = .018
Age, ≥65 years	6/73 626	0.01	
Sex, male	25/151 066	0.02	χ^2^(1) = 1.097, *P* = .295
Sex, female	42/189 033	0.02	
Organic disorders (F00–F09, F70–F79)	0/39 204	0.00	χ^2^(1) = 7.637, *P* = .006
Schizophrenia and related disorders (F20–F29)	5/109 874	0.00	χ^2^(1) = 17.794, *P* < .001
Depressive disorders (F31.3–5, F32–F33)	47/121 763	0.04	χ^2^(1) = 32.918, *P* < .001
Mania (F30, F31.0–2)	0/9517	0.00	*P* = .268
Neurotic and personality disorders (F40–F48, F60–F62)	6/37 231	0.02	χ^2^(1) = 0.107, *P* = .744
Addiction (F10–F19)	6/18 067	0.03	*P* = .171
Others	3/4444	0.07	*P* = .058

Abbreviation: RLS = Restless Legs Syndrome (Fisher’s exact test is used in cases of low expected counts).

### Diagnosis-related differences

Analysis by diagnostic categories (Table 1) indicated that depressive disorders were most commonly associated with RLS, with 47 cases out of 121 763 patients (0.04%) (χ^2^(1) = 32.918, *P* < .001). In contrast, RLS was rarely observed in patients with schizophrenia-related disorders (5/109 874; 0.00%; χ^2^(1) = 17.794, *P* < .001). No cases at all were detected among those diagnosed with organic disorders (0/39 204; 0.00%; χ^2^(1) = 7.637, *P* = .006) or mania (not reaching significance).

### Drug class exposure and RLS occurrence

As seen in Table 2, ADs were the most frequently implicated drug class, accounting for 47 cases among 188 872 exposed patients (0.02%) with an AD being attributed to RLS alone or in combination. Within this category, noradrenergic and specific serotonergic ADs (NaSSA) had the highest proportion of RLS (39/55 843; 0.07%), while few cases were reported for selective serotonin reuptake inhibitors (SSRIs) (2/75 928; 0.00%), serotonin-norepinephrine reuptake inhibitors (SNRIs) (4/52 044; 0.01%), and tricyclic ADs (1/27 622; 0.00%).

**Table 2:** RLS as adverse drug reaction (ADR) related to different classes of psychotropic drugs.

Drug name	Exposed patients (total)	Imputed alone and in combination (W2-W3)	Imputed alone (W2-W3)
Noradrenergic and specific serotonergic antidepressants (NaSSA)	55 843	39 (0.07%)	36 (0.06%)
All antidepressants	188 872	47 (0.02%)	44 (0.02%)
All patients	340 099	67 (0.02%)	64 (0.02%)
Atypical antipsychotics	189 548	22 (0.01%)	19 (0.01%)
All antipsychotics	245 958	23 (0.01%)	20 (0.01%)
Serotonin-norepinephrine reuptake inhibitors (SNRI)	52 044	4 (0.01%)	4 (0.01%)
Other antidepressants	22 134	1 (0.00%)	1 (0.00%)
Tricyclic antidepressants (TCA)	27 622	1 (0.00%)	1 (0.00%)
Selective serotonin reuptake inhibitors (SSRI)	75 928	2 (0.00%)	2 (0.00%)
Typical antipsychotics	108 846	1 (0.00%)	1 (0.00%)

Abbreviations: *n* = number of cases; RLS = Restless Legs Syndrome.

Among antipsychotics (APs), RLS incidence was generally rare, but more common in patients exposed to atypical APs (22/189 548; 0.01%). Only one case among 108 846 exposed to typical APs (0.00%) was imputed alone, and no case imputed in combination, despite the considerable number of exposed patients.

### Specific agents and pharmacological profile

A detailed breakdown of specific medications (Table 3) showed that mirtazapine had the highest absolute number of RLS cases (39/55 843; 0.07%) and was solely responsible for the incidence within the NaSSA group, as mianserine had to be excluded due to the low number of exposed patients. Among SNRIs, venlafaxine was associated with three RLS cases among 37 584 patients (0.01%), while SSRIs such as sertraline (1/19 057) and escitalopram (1/24 790) had even fewer documented cases. Among atypical APs, quetiapine was the most frequently implicated drug (16/63 564; 0.03%), followed by risperidone (4/43 601; 0.01%). Olanzapine was implicated in two cases (2/45 957; 0.00%). Across all ADs and APs, RLS was more commonly imputed alone than in combination. Several drugs across all psychopharmacological groups fulfilled the criterion of ≥5000 cases but were not imputed at all with a “probable” or “definite” rating, including APs (13×), ADs (5×), sedatives (5×), and antiepileptics (4×, for details, see the supplementary material).

**Table 3:** RLS as adverse drug reaction (ADR) related to single substances.

Drug name	Exposed patients	Imputed alone and in combination (W2-W3)	Imputed alone (W2-W3)	NbN pharmacology domain and mode of action
Mirtazapine	54 170	39 (0.07%)	36 (0.07%)	Histamine antagonist (+); norepinephrine, serotonin antagonist (upper dose) (+++)
Quetiapine	63 564	16 (0.03%)	13 (0.02%)	Histamine receptor antagonist (+); norepinephrine reuptake inhibitor and presynaptic receptor antagonist (++); dopamine, serotonin, norepinephrine multimodal (+++)
Amitriptyline	7437	1 (0.01%)	1 (0.01%)	Histamine, serotonin antagonist (+); norepinephrine, serotonin multimodal (+++)
Risperidone	43 601	4 (0.01%)	4 (0.01%)	Dopamine, serotonin antagonist (+); dopamine, serotonin, norepinephrine antagonist (+++)
Venlafaxine	37 584	3 (0.01%)	3 (0.01%)	Serotonin, norepinephrine reuptake inhibitor
Duloxetine	13 853	1 (0.01%)	1 (0.01%)	Serotonin, norepinephrine reuptake inhibitor
Sertraline	19 057	1 (0.01%)	1 (0.01%)	Serotonin reuptake inhibitor
Olanzapine	45 957	2 (0.00%)	2 (0.00%)	Dopamine, serotonin antagonist
Pipamperone	23 293	1 (0.00%)	1 (0.00%)	Not listed
Escitalopram	24 790	1 (0.00%)	1 (0.00%)	Serotonin reuptake inhibitor

The right-most column shows the pharmacological domain and primary mode of action according to the Neuroscience-based Nomenclature (NbN). Where NbN assigns different mechanisms at increasing dose levels, these tiers are marked +, ++, and +++, respectively.

Abbreviations: *n* = number of cases; NbN = Neuroscience-based Nomenclature^13^; RLS = Restless Legs Syndrome.

Table 3 also includes the drug specific pharmacological domain and mode of action as described by the Neuroscience-based Nomenclature.^13^ Notably, the top three highest rating drugs all involve histamine receptor antagonism. Incidence rates (±95% confidence intervals) are provided in Figure 1. The supplementary table provides additional information about the included cases.

**Figure 2:**
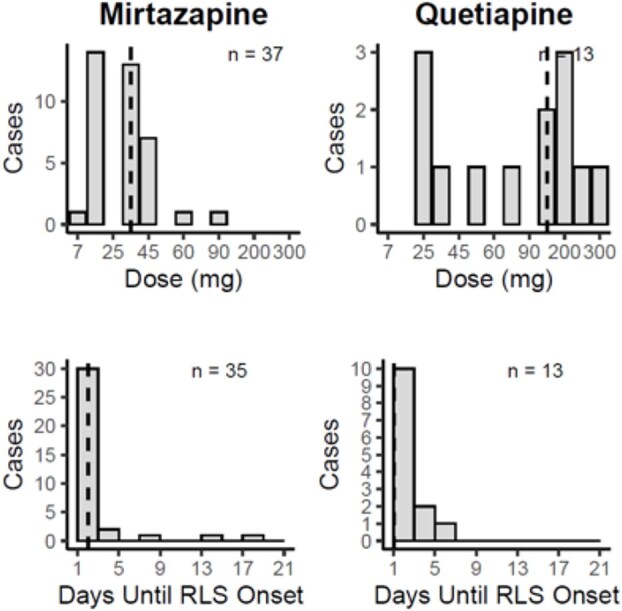
Mirtazapine and quetiapine-associated RLS: Dose distribution and time to onset.

Since mirtazapine and quetiapine stood out the most among all substances, we conducted a more detailed examination of these cases. Interestingly, in 5 (13 %) of the 39 mirtazapine cases, quetiapine was also imputed when lowering the probability rating of the secondary drugs to include “possible” cases. Mirtazapine was even co-imputed in almost half (7/16; 44%) of the quetiapine cases. Drug dosages were generally low at onset of secondary RLS, supporting the potential involvement of histamine receptor antagonism. For mirtazapine, the median prescribed dose was 30 mg (mode: 15 mg; range: 7-90 mg), while for quetiapine the median dose was 100 mg (mode: 25 mg; range: 25-300 mg; full distributions are shown in Figure 2). Slightly more than half of the cases evolved from de novo prescriptions, which expectedly involved lower dosages (mirtazapine: 51.4%; quetiapine: 53.8%). The extended-release formulation of quetiapine (quetiapine XR) was only imputed in 3 of the 16 cases. Most cases exhibited an onset of secondary RLS within 1 or 2 days, with almost no cases evolving after one week of dosage start or change (mirtazapine: median: 2 days; range: 1-18 days; quetiapine: median: 1 day; range: 1-7 days; full distributions are shown in Figure 2).

## Discussion

In summary, real-world evidence from our pharmacovigilance program monitoring over 340 000 inpatients within a 16-year period indicates that severe RLS associated with psychopharmacotherapy is a very rare ADR with 67 detected cases equivalent to a relative frequency of 0.02%. Over 80% of the cases of our sample were attributable to merely two compounds. Regarding ADs, mirtazapine had the highest absolute number of RLS cases compared to all other substances with a total of 39 observations. In the group of APs, low dose quetiapine was found in 16 patients emphasizing its particular role not only in this substance class but also across all observations of secondary RLS in the present study.

Our findings add upon the widely known and accepted paradigm in the literature that delineates the potential of ADs and APs to induce or worsen RLS,^3^^,^^14^^,^^15^ though this remains a very rare phenomenon. Existing investigations dedicated to this topic tend to be heterogenous in terms of study design, patient populations, study duration, or assessment of RLS, leading to conflicting results^4^^,^^6^ that are challenging for clinicians to interpret.

Regarding the impact of ADs to induce RLS, some authors reported large effect sizes for certain substance classes like SSRIs.^16^ Another systematic review reported on a considerable increase of RLS following mirtazapine treatment affecting up to 30% of the patients.^6^ In contrast, a very large Danish study performing a symmetry analysis of 10 000 adults filling in their first SSRI prescription and initiating medication for RLS thereafter came to the conclusion that SSRI treatment and the development of RLS are not associated.^17^ Similarly, a very recent Turkish study comprising 1500 patients found no significant difference between the prevalence of RLS in patients commencing AD treatment compared to healthy controls.^18^ Potential contributors to these discrepancies are elaborated hereinafter. Firstly, some authors point out that many patients may be subject to mild and/or transient symptoms of RLS following AD treatment that usually do not meet clinical significance. This hypothesis is supported by the considerably lower detection of RLS in those studies that did not specifically ask about its symptoms.^6^ Secondly, there is a certain symptom overlap between depression and RLS, especially as far as sleep disturbance and associated consequences like low energy or daytime sleepiness are concerned that may have led to a bias of the results in some studies.^18^^,^^19^ The detected rates of secondary RLS in our investigation approach real-world pharmacovigilance evidence derived from two other studies,^20^^,^^21^ when differences in the observation period are taken into account, which may underscore the validity and clinical significance of the findings.

In the present investigation, mirtazapine was very clearly associated with most cases of drug induced RLS within the substance class of ADs as well as in the overall sample. This result is in line with evidence derived from a systematic review and a large worldwide pharmacovigilance study^6^^,^^21^ as well as an abundance of case reports.^15^ A recent meta-analysis dedicated to this topic in children as well as adults attributed the largest potential to induce RLS to SSRIs/SNRIs, especially fluoxetine and venlafaxine. However, studies with mirtazapine were not included at all in the investigation.^7^ Trazodone, reboxetine, and bupropion were repeatedly discussed as ADs with the lowest potential to induce or worsen RLS,^6^ whereby bupropion was even found to exhibit beneficial or therapeutic effects on RLS in several case reports.^22^

Among APs, low dose quetiapine was associated with the overall largest number of cases of RLS reported in the literature, followed by olanzapine and clozapine.^14^^,^^15^^,^^23^ This is in line with our data, as the majority of quetiapine-imputed cases involved doses well below the AP target range. Considering that APs primarily act by blocking dopamine receptors, and the supposed pathomechanism of RLS involves dopamine dysregulation, the overall low frequency of RLS as an ADR may seem counterintuitive.^3^ However, quetiapine exhibits a dose-dependent mode of action, whereby antihistaminergic, sedating effects are predominantly present in low and midrange dosages. This is also the reason why quetiapine at lower doses is known for its potential as a sleep-promoting drug.^24^ Dosages of at least 300 mg daily are needed to exert a relevant dopaminergic (D2 receptor) and serotonergic (5HT2A receptor) blockade, respectively.^25^^,^^26^ Mirtazapine also exhibits a dose-dependent mode of action. At low dosages it exhibits a predominantly antihistaminergic effect while at higher dosages it enhances serotonergic and noradrenergic neurotransmission.^26^^,^^27^ The frequent co-occurrence of mirtazapine and quetiapine in patients with secondary RLS, with mirtazapine appearing in nearly half of the cases attributed to quetiapine, suggests that the concurrent use of these two psychotropic drugs may have an additive effect contributing to the development of secondary RLS. The low dosages of both mirtazapine and quetiapine as well as their potential additive effect suggests that histamine neurotransmission may represent a crucial common denominator in terms of secondary RLS induced by psychopharmacotherapeutics. Nevertheless, evidence from the literature suggests that other psychotropic as well as nonpsychiatric medications with different pharmacological mechanisms may also contribute to the development or worsening of RLS.^28^

Our study found that, in patients taking mirtazapine or quetiapine, most cases of RLS developed within days of either initiating the medication or increasing the dosage, which is supported by multiple case studies.^22^ Thus, it is highly unlikely that RLS symptoms are caused by a long-term, established psychotropic medication, and other potential causes should be investigated in these cases.

Another reason for the relatively low rates of secondary RLS associated with APs is the potential confusion with akathisia in some patients, due to overlapping symptoms.^4^ In fact, APs differ substantially regarding their potential to cause akathisia with quetiapine revealing a negligible risk,^29^ which makes the hypothesis of misconception in this regard rather unlikely. Further, it is worth mentioning in this regard that substances exhibiting 5-HT2A antagonistic properties, like mirtazapine, may have the potential to ease AP-induced akathisia.^30^ RLS and akathisia are both associated with an urge to move and motor restlessness. Symptoms inherent to RLS include the presence of a circadian rhythm and uncomfortable sensations usually in the legs that are relieved by movement. Inner restlessness, however, may be diagnostic of akathisia.^4^

The strengths of the current investigation comprise a very structured and consistent documentation process of ADRs in a real-world setting with several checkpoints in terms of quality control within participating hospitals, regional conferences, and the AMSP headquarters. This procedure enhances the quality of the collected data and lacks several problems inherent to retrospective studies in general. The long observation period enables the detection of very rare events with sufficient precision. In contrast to studies including outpatient populations, we could rely on verified exposures of psychotropic drugs due to the direct administration of medication by nursing staff rather than a mere prescription of a drug. The most relevant limitation of AMSP is underreporting of ADRs, which is a known and crucial limitation of pharmacovigilance studies in general. Physicians who serve as drug monitors generally do this alongside their clinical work. Despite individual motivation and training of the participating staff that in turn was found to be a protective factor regarding underreporting of ADRs,^31^ personal time as well as the financial resources of the participating hospital may be relevant factors that potentially contribute to an individual and/or institutional bias. In addition, specifically in psychiatric patients, polypharmacy and the attribution of somatic symptoms to the underlying psychiatric disorder may further contribute to underrecognition.^32^ Further, generalizability of our results for outpatient populations is limited.

In conclusion, our results add upon existing knowledge that clinically relevant severe secondary RLS associated with ADs or APs in real-world inpatient clinical routine settings seems to be a rare phenomenon, whereby substances that exert antihistaminergic properties should be particularly considered. Discrepancies regarding the prevalence of RLS associated with psychopharmacotherapy in some clinical studies, especially as far as ADs are concerned, may partly be explained by a symptom overlap with depression as such or subthreshold/transient RLS manifestations that remained below the detection threshold of our study protocol.

## Supplementary Material

Supplement_ID-25-0210_pyag029

## Data Availability

The data that support the findings of this study are available from the corresponding author upon reasonable request.
