# Supplementary material for: Secondary Restless Legs Syndrome during psychopharmacological treatment: real-world evidence from a multinational pharmacovigilance program
Source: Int J Neuropsychopharmacol. 2026 Jun 7;29(7):pyag029. doi: 10.1093/ijnp/pyag029 (PMC13326631; doi:10.1093/ijnp/pyag029)
Supplement: Supplement_ID-25-0210_pyag029 [file supplement_id-25-0210_pyag029.docx]

Title: Secondary Restless Legs Syndrome during Psychopharmacological Treatment: Real-World Evidence from a Multinational Pharmacovigilance Program

Supplementary material 1: Comprehensive list of included cases of psychopharmacologically induced RLS

| **Nr.** | **ICD-10 Dg** | **Medication 1^1^** | **Med 1 dose (mg)** | **Med 1 probability^2^** | **Medication**  **other^1^** | **Med other dose (mg)** | **Med other probability^2^** | **DoE ^3^**  **(days)** | **Mode of exposure** | **Notes** |
| --- | --- | --- | --- | --- | --- | --- | --- | --- | --- | --- |
| 1 | F53.1 | amitriptyline | 100 | 2 | citalopram | 10-30 | 1 | 1 | Start  100 mg | ADR remitted 1 day after reduction of amiriptyline to 25mg;  possible additive effect of citalopram |
| 2 | F33.1 | escitalopram | 5 | 2 |  |  |  | 3 | Start  5 mg | ADR had occurred in the past under paroxetine and sulpirid |
| 3 | F33.2, F13.2 | sertralin | 100 | 3 |  |  |  | 2 | Increase  50 to 100 mg | Definite ADR, since it had occurred before under sertraline |
| 4 | F33.1 | mirtazapine | 45 | 2 |  |  |  | 1 | Increase  30 to 45 mg | ADR remitted 1 day after mirtazapine disontinuation |
| 5 | F33.2, F10.1 | mirtazapine | 30 | 2 |  |  |  | 1 | Start  30 mg | ADR remitted 1 day after mirtazapine discontinuation |
| 6 | F33.2 | mirtazapine | 30 | 2 | venlafaxine | 225-300 | 1 | 3 | Increase  15 to 30 mg | venlafaxine exposure for weeks, possible additive effect  ADR remitted after mirtazapine discontinuation not reduction |
| 7 | F32.2 | mirtazapine | 15-45 | 2 |  |  |  | 1 | Start  15 mg | Polysomnography performed: PLMS;  ADR remitted 5 days after mirtazapine discontinuation |
| 8 | F33.2, F10.2 | mirtazapine | 15 | 2 |  |  |  | 1 | Start  15 mg | Possible polyneuropathy;  ADR remitted 1 day after mirtazapine discontinuation |
| 9 | F32.2, F10.1 | mirtazapine | 30 | 2 |  |  |  | 1 | Start  30 mg | ADR remitted 1 day after mirtazapine discontinuation |
| 10 | F33.2 | mirtazapine | 60 | 2 | amitriptyline | 75-150 | 1 | 1 | Increase  45 to 60 mg | Mirtazapine 30-45 mg for 3 months, ADR persisted after reduction to 30 mg, remitted 2 days after discontinuation |
| 11 | F33.1 | mirtazapine | 15 | 2 |  |  |  | 1 | Start  15 mg | ADR remitted promptly after mirtazapine discontinuation |
| 12 | F32.2 | mirtazapine | 30 | 2 |  |  |  | 3 | Start  30 mg | Subclinical RLS without insomnia worsened under mirtazapine;  ADR remitted 5 days after reduction to 15 mg |
| 13 | F33.2 | mirtazapine | 45 | 2 |  |  |  | 3 | Increase  30 to 45 mg | ADR remitted 2 days after mirtazapine disontinuation |
| 14 | F32.2 | mirtazapine | 30 | 2 | paroxetine | 20 | 1, 5 | 8 | Increase  15 to 30 mg | No symptoms in legs, only arms, possibly discrete idiopathic RLS; ADR remitted promptly after mirtazapine discontinued |
| 15 | F32.2 | mirtazapine | 15-30 | 2 |  |  |  | 15 | Start  15 mg | Polysomnography performed: PLMS;  ADR remitted 1 day after mirtazapine discontinuation |
| 16 | F32.2 | mirtazapine | 15 | 2 |  |  |  | 1 | Start  15 mg | Relief with movement not described;  ADR remitted promptly after discontinuation |
| 17 | F51.0 | mirtazapine | 7,5 | 2 |  |  |  | 1 | Start  7,5 mg | Polysomnography performed: PLMS;  ADR remitted promptly after mirtazapine discontinuation |
| 18 | F33.2 | mirtazapine | 15 | 2 | sertraline | 25-50 | 1 | 3 | Start  15 mg | Comorbid benzodiazepine withdrawal: not clinically relevant;  ADR remitted promptly after mirtazapine discontinuation |
| 19 | F23.0 | mirtazapine | 15 | 2 | quetiapine | 300 | 1 | 1 | Start  15 mg | quetiapine and pipamperone for more than 2 months;  ADR remitted 1 day after mirtazapine discontinuation |
| 20 | F32.2 | mirtazapine | 90 | 2 | quetiapine  escitalopram | 250-400  30 | 1 | 2 | Increase  60 to 90 mg | ADR remitted 3 days after mirtazapine reduction to 60 mg |
| 21 | F33.2 | mirtazapine | 45 | 2 |  |  |  | 20-30 | Increase  30 to 45 mg | Starting date of ADR unclear;  ADR remitted 6 days after mirtazapine discontinuation |
| 22 | F33.2 | mirtazapine | 45 | 2 | venlafaxine | 75-150 | 1 | 1 | Increase  30 to 45 mg | ADR remitted 1 day after mirtazapine reduction to 15 mg |
| 23 | F33.2, F10.2 | mirtazapine | 45 | 2 |  |  |  | 30-40 | Increase  30 to 45 mg | ADR remitted 1 day after mirtazapine discontinuation, not after reduction |
| 24 | F32.1 | mirtazapine | 45 | 2 | metoclopramide | 5 | 5 | 1 | Increase  30 to 45 mg | ADR remitted 3 days after reduction to 30 mg;  possible interaction with metoclopramide (inhibitor of CYP 2D6) |
| 25 | F32.2, F10.2 | mirtazapine | 15-30 | 2 |  |  |  | 3 | Start  15 mg | ADR remitted 1 day after mirtazapine discontinuation |
| 26 | F32.1 | mirtazapine | 45 | 2 | quetiapine XR | 100 | 1 | 3 | Increase  30 to 45 mg | ADR remitted 2 days after mirtazapine discontinuation |
| 27 | F32.2, F41.1 | mirtazapine | 15 | 2 | venlafaxine  quetiapine | 150  300 | 1  1 | 1 | Start  15 mg | ADR started and stopped with mirtazapine |
| 28 | F41.1 | mirtazapine | 30 | 3 | quetiapine XR | 250 | 1 | 2 | Start  30 mg | ADR remitted 1 day after mirtazapine discontinuation; |
| 29 | F32.2 | mirtazapine | 30 | 2 |  |  |  | 1 | Increase  15 to 30 mg | ADR remained after dosage reduced to 15 mg, remitted 1 day after discontinuation |
| 30 | F33.1 | mirtazapine | 15 | 2 |  |  |  | 1 | Increase  15 to 30 mg | ADR remitted the day after mirtazapine was discontinued |
| 31 | F32.3 | mirtazapine | 30 | 2 | quetiapine | 150 | 2 | 2 | Increase  15 to 30 mg | No ADR under quetiapine alone |
| 32 | F33.2 | mirtazapine | 15 | 2 |  |  |  | 5 | Start  15 mg | ADR remitted the day after mirtazapine was discontinued |
| 33 | F33.1 | mirtazapine | 30-45 | 3 |  |  |  | 2 | Increase  15 to 30 mg |  |
| 34 | F32.2 | mirtazapine | 30 | 2 |  |  |  | 2 | Increase  15 to 30 mg | ADR remitted the day after mirtazapine was discontinued |
| 35 | F60.3,  F33.1 | mirtazapine | 30 | 2 |  |  |  | 2 | Increase  7,5 to 15 mg | ADR remitted 3 days after mirtazapine was discontinued |
| 36 | F33.2 | mirtazapine | 15 | 3 |  |  |  | 4 | Start  15 mg | Definite ADR, since it had occurred before under mirtazapine monotherapy |
| 37 | F43.2 | mirtazapine | 30 | 2 |  |  |  | 2 | Increase  15 to 30 mg |  |
| 38 | F10.2 | mirtazapine | 15 | 2 | amitriptyline | 25 | 1 | 3 | Start  15 mg | ADR remitted the day mirtazapine was discontinued |
| 39 | F33.2 | mirtazapine | 15 | 2 |  |  |  | 18 | Start  15 mg |  |
| 40 | F23.2 | mirtazapine | 30 | 2 |  |  |  | 2 | Start  30 mg | ADR remitted 2 days after mirtazapine was discontinued |
| 41 | F33.3, F10.1 | agomelatine | 25 | 2 | olanzapine | 10 | 1 | 4 | Start  25 mg | ADR remitted 2 days after agomelatine was discontinued |
| 42 | F43.2, F63.0 | venlafaxine | 150 | 2 | melperone | 50 | 5 | 1 | Increase  75 to 150 mg |  |
| 43 | F32.2 | venlafaxine | 150 | 2 |  |  |  | 14 | Increase  150 to 225 mg | No remission of the ADR after reduction to 150 or later 75 mg but 2 days after discontinuation |
| 44 | F32.1, F40.01 | venlafaxine | 300 | 2 |  |  |  | 1 | Increase  225 to 300 mg | ADR pre-existing; worsening and clinical significance after dosage increase; thereafter switch to duloxetine leading to remission of the ADR after 3 weeks |
| 45 | F33.2 | Duloxetine | 90 | 3 |  |  |  | 4 | Increase  60 to 90 mg | At given point, no cases known for duloxetine-induced RLS;  ADR remitted 1 day after reduction to 60 mg |
| 46 | F32.2 | Olanzapine | 20 | 2 | mianserine  levomepromazin | 120  50 | 1  5 | 17 | Increase  15 to 20 mg | ADR remitted 3 days after olanzapine discontinuation;  Additional EPMS possible, biperiden was administered; High blood levels of olanzapine as a result of interaction with levomepromazine |
| 47 | F20.0 | Olanzapine | 20 | 2 |  |  |  | 1 | Start  20 mg | ADR remitted 3 days after switch to clozapine |
| 48 | F13.2 | Quetiapine | 25-50 | 2 | escitalopram  risperidone | 20  0,5-2 | 1 | 1 | Start  25 mg | ADR remitted promptly after quetiapine discontinuation |
| 49 | F32.2 | Quetiapine | 200 | 3 | mirtazapine  escitalopram | 15-45  20 | 1  1 | 4 | Increase  100 to 200 mg | ADR remitted after reduction of quetiapine to 100 mg;  ADR reappeared after re-exposition to 200 mg |
| 50 | F50.0, F32.1 | quetiapine | 200 | 2 | mirtazapine | 30 | 1 | 1 | Increase  150 to 200 mg | ADR started when mirtazapine was administered,  on the same day quetiapine raised from 150 to 200 mg;  remission after quitting of quetiapine, mirtazapine was not changed |
| 51 | F32.2 | quetiapine | 225-300 | 2 | venlafaxine  metoprolol | 150-225  47,5 | 1  5 | 1 | Increase  175 to 225 mg | ADR remitted 4 days after quetiapine discontinuation |
| 52 | F60.3 | quetiapine | 75-100 | 3 | duloxetine  tramadol | 90  300 | 1  1 | 2 | Increase  50 to 75 mg | ADR had occurred repeatedly under quetiapine; RLS treated with pramipexole, quetiapine not discontinued |
| 53 | F19.2, F32.2 | quetiapine | 300, 200 | 3 | mirtazapine | 45 | 1 | 1 | Increase  150 to 300 mg | ADR remitted after quetiapine reduction to 100 mg,  reappeared with 200 mg, remitted when quetiapine paused;  mirtazapine at stable dosage, possible additive effect |
| 54 | F19.2 | quetiapine | 50 | 2 | venlafaxin | 75-150 | 1 | 7 | Start  50 mg | ADR remitted the day after quetiapine was discontinued |
| 55 | F60.3 | quetiapine | 25-50 | 2 | venlafaxin | 150 | 1 | 1 | Start  25 mg | ADR persisted after dose increase;  ADR remitted 2 days after quetiapine was discontinued |
| 56 | F60.3 | quetiapine | 25 | 2 | mirtazapine | 15 | 1 | 1 | Start  25 mg | ADR remitted the day after quetiapine was discontinued |
| 57 | F25.2 | quetiapine | 100 | 2 | olanzapine | 15-30 | 1 | 1 | Start  100 mg | ADR remitted the day after quetiapine was discontinued |
| 58 | F31.3 | quetiapine | 100 | 2 | quetiapine XR | 200 | 2 | 2 | Increase | ADR occurred after simultaneous increase of 50 to 100 mg (IR) and 100 to 200 mg (XR), remission after both were decreased |
| 59 | F33.2 | quetiapine | 100, then later 25 | 3 |  |  |  | 1 | Start 100 mg and 25 mg | Definite ADR, since it occurred again under quetiapine |
| 60 | F33.2, F43.1 | quetiapine | 200-300 | 2 | mirtazapine | 30 | 2 | 4 | Start  30 mg | ADR improved but did not remit after mirtazapine was discontinued, remission after quetiapine was also discontinued |
| 61 | F33.2 | quetiapine XR | 200-300 | 2 | mirtazapine | 15 | 2 | 2 | Increase  100 to 200 mg | ADR improved but did not remit after mirtazapine was discontinued, remission after quetiapine was also discontinued |
| 62 | F33.2 | quetiapine XR | 50 | 2 | mirtazapine  fluoxetine | 30-45  20 | 1  1, 5 | 1 | Start  50 mg | ADR started on the day both quetiapine XR and mirtazapine were administered, stopped after quetiapine XR was discontinued; fluoxetine: additive and pharmacokinetic effect on mirtazapine |
| 63 | F33.3 | risperidone | 3 | 2 | escitalopram | 20 | 1 | 1 | Increase  2 to 3 mg | ADR remitted 3 days after risperidone reduction to 1 mg  possible additive effect of escitalopram; |
| 64 | F20.0 | risperidone | 4 | 2 |  |  |  | 20 | Increase  3 to 4 mg | ADR remitted after reduction to 1 mg |
| 65 | F33.2, F00.1 | risperidone | 2 | 2 | amitriptyline | 50 | 1 | 3 | Increase  1 to 2 mg | ADR remitted 6 days after risperidone was discontinued |
| 66 | F33.2, F41.1 | risperidone | 0,5 | 2 | agomelatine | 12,5 | 1 | 1 | Start  0,5 mg | ADR remitted the day after risperidone was discontinued |
| 67 | F11.2, F33.9 | pipamperone | 20 | 2 | venlafaxine | 75 | 1 | 1 | Start  20 mg | ADR remitted after discontinuation of pipamperone |

**^1^** **Medication 1**: Main drug associated with secondary RLS; **Medication other**: other drugs associated with secondary RLS

**^2^ 1: possible** ADR (ADR not known for the drug, time course or dosage are unusual, or an alternative explanation is seen as more probable.

**2: *probable*** ADR (ADR known for the drug, the time course and dosage are in accordance with previous experience, and alternative explanations are less likely.

**3:** ***definite*** *(*the criteria necessary for “probable” are fulfilled and a re-appearance of the ADR occurs after re-exposure to the suspected drug

**4:** ***questionable*** *(grade 4a)* ADR or **case not sufficiently documented** (grade 4b).

**5:** **drug interaction possible** (pharmacokinetic drug interactions are not known for the suspected drug or known interactions have not been evaluated by drug plasma levels)

**6:** ***drug interaction definite*** *(*documented plasma levels are reflecting said interaction).

**^3^ DoE = Duration of exposure** in days: time from initiation of suspected drug (medication 1) to emergence of ADR; in case a dosage increase was suspected to have caused the ADR, DoE states the time from the dosage increase to the emergence of RLS

Supplementary material 2: List of psychopharmacotherapeutics with no probable (grad 2) or definite (grade 3) ratings

The following drugs with >= 5000 exposed cases yielded no imputations with a probable or definite rating: amisulpride (n = 12,748), aripiprazole (n = 15,442), biperiden (n = 20,692), carbamazepine (n = 11,336), chlorprothixene (n = 8,811), citalopram (n = 20,085), clozapine (n = 24,207), diazepam (n = 24,882), doxepin (n = 5,772), flupentixol (n = 7,015), haloperidol (n = 21,060), lamotrigine (n = 11,382), levomepromazine (n = 8,166), lorazepam (n = 67,171), melperone (n = 12,234), oxazepam (n = 10,868), paroxetine (n = 5,257), perazine (n = 5,549), pipamperone (n = 18,842), pregabalin (n = 12,541), promethazine (n = 11,236), prothipendyl (n = 13,469), trazodone (n = 11,497), trimipramine (n = 8,297), valproic acid (n = 36,924), zolpidem (n = 11,597), zopiclone (n = 14,467), zuclopenthixol (n = 5,787, alphabetical order).
